# Cutaneous Melioidosis Cluster Caused by Contaminated Wound Irrigation Fluid

**DOI:** 10.3201/eid2208.151149

**Published:** 2016-08

**Authors:** Adam J. Merritt, Mariani Peck, Dionne Gayle, Avram Levy, Yi-Horng Ler, Edward Raby, Tristan M. Gibbs, Timothy J.J. Inglis

**Affiliations:** PathWest Laboratory Medicine, Nedlands, Western Australia, Australia (A.J. Merritt, M. Peck, D. Gayle, A. Levy, Y.-H. Ler, T.M. Gibbs, T.J.J. Inglis);; University of Western Australia, Crawley, Western Australia, Australia (A.J. Merritt, T.J.J. Inglis);; Royal Perth Hospital, Perth, Western Australia, Australia (E. Raby)

**Keywords:** Burkholderia pseudomallei, disease outbreaks, health facilities, hygiene, microscopy, electron, scanning, multilocus sequence typing, soft tissue infections, Western Australia, bacteria, bacterial infections, contamination, saline, wound irrigation

## Abstract

Melioidosis usually occurs after environmental exposure to *Burkholderia pseudomallei* in the tropics. A cluster of 5 cutaneous melioidosis cases occurred in suburban southwest Australia after an earlier case in January 2012. We collected environmental samples at the first patient’s home in January 2012 and from a nearby health center in December 2013 after 2 new cases occurred in the same postal district. We isolated genotypically identical *B. pseudomallei* from the first patient and 5 other patients in the district. Environmental sampling implicated an opened bottle of saline wound irrigation fluid containing >10^6^ *B. pseudomallei*/mL. The bottle included instructions to discard within 24 hours of opening. No further cases of *B. pseudomallei* infection occurred after removing the contaminated bottle. This cutaneous melioidosis cluster demonstrates that *B. pseudomallei* can survive and disseminate in widely used medical fluids beyond its known geographic distribution, highlighting a need to use these products according to manufacturers’ instructions.

## Abstract 2

*Burkholderia pseudomallei* can cause healthcare-associated infections outside its recognized tropical zone.

Melioidosis, caused by infection with the bacterium *Burkholderia pseudomallei*, is a disease with manifestations ranging from rapidly fatal septicemia, pneumonia, or meningoencephalitis to localized abscess formation, cellulitis, and asymptomatic seroconversion. This disease occurs most commonly in Southeast Asia and northern Australia after exposure to contaminated soil or surface water (*1*). The US National Notifiable Diseases Surveillance Systems case definition describes cutaneous melioidosis as “an acute or chronic localized infection which may or may not include symptoms of fever and muscle aches. Such infection often results in ulcer, nodule, or skin abscess” (*2*). 

Sporadic cases outside melioidosis-endemic regions usually occur in persons who have a history of travel in the tropics, which can be as long as several decades previously because of the ability of *B. pseudomallei* to persist undetected after the initial inoculation event (*3*). In these cases, *B. pseudomallei* infection might not be considered in the differential diagnosis. Detection of sporadic cases of melioidosis by clinical pathology laboratories requires microbiology laboratories to have robust bacterial identification procedures. Even with advanced equipment, a lack of awareness of the characteristic features of *B. pseudomallei* can result in misidentification of cultured organisms (*4*). Only a few point-source outbreaks of melioidosis have been reported (*5*). Two of these occurred in Western Australia; 1 was attributed to movement of livestock from the tropical north to the temperate southwest (*6*), and the other was caused by contamination of a potable water supply (*7*). Neither cluster was healthcare-associated. Only a few cases of healthcare-associated melioidosis have been reported. Some of the earliest accounts of melioidosis identified opiate injection as a potential source of infection (*8*). In animal healthcare, injected medication was thought responsible for a series of animal infections in northern Australia (*9*). The first report of hospital-acquired melioidosis originated in Hawaii, USA, and described pulmonary infection after bronchoscopy with a scope contaminated with *B. pseudomallei* (*10*). This report indicated that the contaminated bronchoscope had previously been used on a returned traveler with melioidosis. A second report described 2 patients with *B. pseudomallei* urinary tract infection on different wards of a hospital on whose grounds *B. pseudomallei* was isolated (*11*). Nosocomial contamination associated with faulty hospital hygiene and ineffective disinfectant solution was reported from a hospital in Thailand treating patients with melioidosis (*12*). More recently, cases of neonatal melioidosis from a hospital in Thailand were thought to be healthcare-associated, although the full details of transmission could not be determined (*13*). 

Melioidosis became a notifiable infection in Western Australia in January 2000 (*14*). Physicians, pathology service providers, and the state public health laboratory are required to report a diagnosis of melioidosis to the State Disease Control Directorate. Melioidosis notification is largely laboratory-generated in Western Australia because confirmation of infection according to the Australian Laboratory Case Definition relies on culture of *B. pseudomallei* from clinical specimens. Pathology service providers therefore routinely refer presumptive *B. pseudomallei* isolates to the state public health laboratory for confirmation, genotyping, and archiving in a reference culture collection (Western Australian *Burkholderia* Collection). Here we report the laboratory investigation of a cluster of cutaneous melioidosis in the temperate southwest of Australia, the identification of its source, and means of control.

## Methods

In January 2012, a patient residing in temperate Western Australia who had a superficial soft tissue infection had a preliminary isolation of *B. pseudomallei*. We interviewed the patient to determine a detailed local, national, and international travel history; potential means of *B. pseudomallei* exposure; and melioidosis-associated concurrent conditions. 

The patient’s home property was visited by staff from PathWest Laboratory Medicine (Nedlands, Western Australia) for inspection and environmental sampling of garden beds (1 sample), potable water (2 samples), storm water drainage (1 sample), and a nearby nature reserve (1 sample). Soil samples were processed by suspending 10 g of soil in 20 mL of sterile water and incubated overnight with agitation. Samples were kept stationary for 2 h to allow the soil to settle, and 50 μL supernatant was spread across Ashdown’s agar and *B. pseudomallei* selective agar (*15*,*16*). Solid media was incubated for 48 hours at 37°C followed by 5 days at room temperature. One milliliter of supernatant was also inoculated into 10 mL of Ashdown’s broth (*17*) and incubated at 37°C overnight before being spread onto selective solid media as described. Plates were checked every 24 h and suspect colonies picked to nonselective blood agar with a 10-μg gentamicin disk placed on the second sector.

In December 2013, after 2 additional cases of culture-confirmed cutaneous melioidosis had been detected in the same postal district associated with a local healthcare facility, we conducted environmental sampling in and around the facility. Additional environmental samples were collected from public accessed land and building excavations in the neighborhood to identify other potential sources of *B. pseudomallei* external to the health facility. These samples were processed as described previously. Samples included all wound care products in current use, whether sealed or already open. We also sampled fixed surfaces patients were likely to come into contact with during wound care and soil at locations surrounding the health facility that could provide either a primary source or reservoir for later distribution. Laboratory-based surveillance for melioidosis cases from the region was performed for 12 months after the conclusion of the field investigation. This process included referral of all suspected *B. pseudomallei* by all pathology service providers in Western Australia, multilocus sequence typing (MLST) of all confirmed *B. pseudomallei*, and matrix-assisted laser desorption/ionization time-of-flight (MALDI-TOF) mass spectrometry identification of all wound swab bacterial isolates processed at the state public health laboratory (*4*).

Solid samples were inoculated onto 5% horse blood agar, Ashdown’s selective agar, and *Burkholderia* selective agar and then incubated for 48 h at 37°C. Bulk liquids were sampled in a class II biological safety cabinet not previously used for *B. pseudomallei* work, and 1.0 mL dispensed into thioglycollate broth in accordance with standard microbial contamination assessment methods. A ≈100-mL aliquot of each bulk liquid was filtered through a 0.22-µm membrane filter and used to inoculate the same series of selective and nonselective agars. Positive fluid samples were used for detailed bacterial count studies by using a serial 10-fold dilution to 1:10^7^, a spiral plating device, and triplicates of each dilution.

One environmental sample yielded *B. pseudomallei*: a 1,000-mL bottle of wound irrigation fluid. Turbidity of the residual wound irrigation fluid in the bottle was not visible because the container walls were semi-opaque. To determine the extent of bacterial colonization and identify any specific higher-density bacterial localization within it, the bottle was dissected from its screw top down to its bottom, sampling at 2.5-cm intervals. Plastic surfaces contaminated with *B. pseudomallei* were cut into ≈0.5-cm square portions and processed for scanning electron microscopy. Samples were fixed overnight (2.5% glutaraldehyde [vol/vol] in 0.05 mol/L cacodylate buffer pH 7.4), then washed in the same buffer before postfixation for 30 min (1% aqueous osmium tetroxide) and sequential dehydration in ethanol series for 5 min each. Reagents were supplied by the PathWest Electron Microscopy Unit (Nedlands, Western Australia). Critical point drying was achieved by using liquid carbon dioxide; the plastic squares were then attached to aluminum stubs by using double-sided carbon tape with edges painted with carbon solution and coated with 15 nm of carbon. Samples were viewed through a Zeiss SUPRA 55 Variable Pressure SEM operating at 3–5 kV with either in-lens or SE2 detectors, depending on magnification required.

Preliminary identification of *B. pseudomallei* was made by using a MALDI-TOF mass spectrometer with a 70% formic acid partial extraction protocol and a locally generated *Burkholderia* mass spectrum database (*4*). Definitive confirmation of *B. pseudomallei* was made by using a panel of real-time PCR assays targeting independent genes (*18*,*19*). The mutually exclusive *B. thailandensis*–like flagellar (BTFC)/*Yersinia*-like fimbrial (YLF) genetic markers were used for preliminary molecular characterization, which used primers and probes developed in-house based on the previously published sequences (*20*). MLST was performed by using the current PCR and sequencing primers as previously described (http://bpseudomallei.mlst.net/misc/info2.asp) (*21*). Sequencing was performed on an ABI 3130xl sequencer by using forward and reverse primers with ABI BigDye version 3.1 sequencing chemistry (Applied Biosystems, Foster City, CA, USA). MEGA5 was used to construct a neighbor-joining tree of MLST sequence from all outbreak-associated isolates, including the wound irrigation fluid isolate, to show their genetic relationship to reference *B. pseudomallei* isolates in the Western Australian *Burkholderia* Collection (*22*,*23*). *B. thailandensis* E264 was used as an outgroup and root for the neighbor-joining tree. Bootstrap values >50 (>1,000 replicates) were included next to the tree’s branches (*24*), and evolutionary distances were computed by using a maximum composite likelihood method with units of the number of base substitutions per site (*25*). The rate variation among sites was modeled with a gamma distribution (shape parameter = 4).

## Results

### Case Summary

The state public health laboratory started its investigation in 2012 after confirming a diagnosis of cutaneous melioidosis in a patient who had not left the temperate southwest region of Western Australia during the previous 18 years (patient A). No further cases of cutaneous melioidosis occurred throughout 2012. In September 2013, an isolate of suspected *B. pseudomallei* was referred from another resident of the same postal district (patient C) who had a purulent wound infection at the site of a minor procedure 1 month before. We commenced more intensive public health investigations after a further case of culture-confirmed cutaneous melioidosis (discharge at site of leg injury [patient D]) and prioritized referral of all suspected *B. pseudomallei* isolates from pathology service providers in Western Australia. Four further cases occurred after this time (patients E through H). An additional 2012 case was detected (patient B) after retrospective review. *B. pseudomallei* isolates were available from a total of 8 cases (Table). We excluded 2 cases (patients B and H) from further investigation of the cluster after genotyping yielded sequence types (STs) already documented in clinical and environmental isolates from Southeast Asia (ST-84 and ST-176) and both were shown to be YLF types (Table). These 2 patients had different clinical features and a history of travel to a known melioidosis region. 

**Table:** Summary characteristics of a cutaneous melioidosis cluster caused by contamination of wound irrigation fluid, Western Australia, 2012–2013*

Isolate source	Source	Date of collection	Pathology request notes	MALDI-TOF MS score	YLF/BTFC	MLST ST
Patient A	Wound	2012 Jan 20	Cellulitis (left shin and toe), unresponsive to first-line antibiotics	2.6	BTFC	1112
Patient B	Pulmonary	2012 Mar 30	Cough and shortness of breath, fine needle aspirate of lung lesion, fever and chills afterwards	2.476	YLF	84
Patient C	Wound	2013 Sep 25	Purulent, dehiscing wound at site of lesion removed 1 month previously	2.61	BTFC	1112
Patient D	Wound	2013 Nov 12	Leg injury, slough	2.376	BTFC	1112
Patient E	Wound	2013 Nov 29	Cellulitis (left shin), worsening despite first-line antibiotics	2.146	BTFC	1112
Patient F	Wound	2013 Dec 05	Nonhealing wound (right forearm)	2.394	BTFC	1112
Patient G	Wound	2013 Dec 13	Wound sustained in QLD, swabbed to check for cutaneous melioidosis	2.7	BTFC	1112
Patient H	Pulmonary† and cutaneous	2013 Dec 20	Subgaleal abscess pus	2.211	YLF	176
Saline	Wound irrigation fluid	2013 Dec 20	NA	2.3	BTFC	1112

*Patients ordered by date of specimen collection. Isolates from patients B and H have sequence types previously documented in Southeast Asia. BTFC, *B. thailandensis*–like flagellar gene cluster; MALDI-TOF MS, matrix-assisted laser desorption/ionization time-of-flight mass spectrometry; MLST, multilocus sequence typing; NA, not applicable; QLD, Queensland; ST, sequence type; YLF, *Yersinia*-like fimbrial gene cluster. †Molecular detection only; no isolate recovered.

The patient from January 2012 and all subsequent patients with cutaneous infections associated with the common genotype had minor wounds dressed at a health facility in the same postal district. One patient (patient G) had a traumatic wound swabbed to check whether cutaneous melioidosis was present and thus might represent a case of contamination or colonization. Apart from patients B and H, all other patients were reported to have local skin inflammation or cellulitis with or without discharge; infection in 3 patients had not responded to presumptive antimicrobial therapy.

### Isolate Characterization

**Figure 1:**
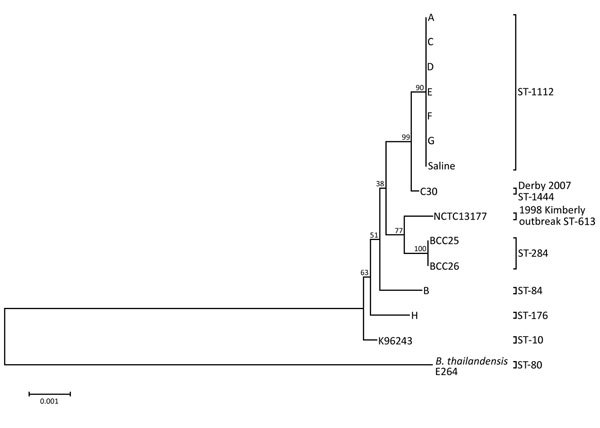
Neighbor-joining tree of aligned multilocus sequence typing sequences of *Burkholderia pseudomallei* clinical isolates from a 2012–2013 cutaneous melioidosis cluster in the temperate southern region of Western Australia (patients A and C–G) and indistinguishable environmental isolate (saline) with sequence type (ST) 1112 and their genetic relatedness to other isolates from the Western Australian *Burkholderia* Collection (C30, NCTC13177, BCC25, and BCC26). Isolates from patients B and H are shown as less closely related to the ST-1112 cluster. *B. thailandensis* E264 is used as an outgroup and root for the tree. Tree inference was performed in MEGA5 (*22*). Bootstrap values >50 (>1,000 replicates) are shown. Scale bar indicates base substitutions per site (*20*–*24*).

Our state public health laboratory confirmed the identity of all isolates by MALDI-TOF mass spectrometry and a real-time PCR assay panel. Specimens from the January 2012 patient and all subsequent patients with cutaneous melioidosis yielded cultures of *B. pseudomallei* belonging to the BTFC clade of *B. pseudomallei* (Table). Bacterial isolates belonged to a single MLST genotype, previously unreported in the global *B. pseudomallei* database (Figure 1). For this genotype, the *gltB* locus had 1 single nucleotide polymorphism variant (G276A) of *gltB* allele 16, given allele number 85, and a new ST, ST-1112. The closest related strains in the *B. pseudomallei* MLST database are all double-locus variants of ST-1112 and are all identified as Australian human or veterinary clinical isolates. Comparison with clinical and environmental isolates from Western Australia identified a previous clinical *B. pseudomallei* isolate (C30) that varied at a single locus, from the town of Derby in the north of the state in 2007 (Figure 1).

### Environmental Sampling

**Figure 2:**
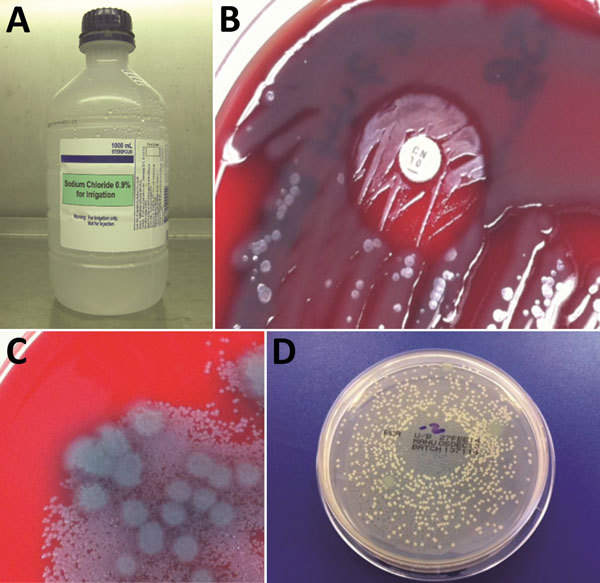
Bacterial culture results for 1,000-mL bottle of wound irrigation fluid in laboratory investigation of a 2012–2013 cutaneous melioidosis cluster in the temperate southern region of Western Australia. A) Wound irrigation fluid in original bottle. B) Direct primary culture of wound irrigation fluid on blood agar plate, showing growth inhibition of *Pseudomonas aeruginosa* and revealing *Burkholderia pseudomallei* around gentamicin disk. C) Filtrate of wound irrigation fluid from same bottle showing higher count of *B. pseudomallei* colonies than *P. aeruginosa*. D) Dilution of wound irrigation fluid (1:100), dispensed by spiral plating device, showing *B. pseudomallei* colonies and relatively sparse *P. aeruginosa* colonies.

Environmental sampling followed a spiral plan, beginning in the health facility and working outwards into the grounds and wider neighborhood, based on potential for public exposure. This process generated a total of 62 samples, including surface swabs, wound dressing materials, fluids, ointments, creams, garden soil, and building site soil. The bottle of wound irrigation fluid that yielded *B. pseudomallei* had been in intermittent use since September 2013 (Figure 2, panels A and B). PCR assays and MLST performed on the wound irrigation fluid isolate confirmed the presence of *B. pseudomallei* BTFC/ST-1112, matching the clinical isolates from the patients with cutaneous melioidosis. Another previously opened bottle of wound irrigation fluid and unopened bottles from the same supplier batch all were culture-negative for *B. pseudomallei*. However, other fluid samples, including in-use disinfectants, grew small quantities of *Pseudomonas aeruginosa*, which was also present in the *B. pseudomallei*–contaminated wound irrigation fluid bottle. The contaminated wound irrigation fluid contained 1.83 × 10^6^ CFU/mL *B. pseudomallei* and 1.89 × 10^3^ CFU/mL *P. aeruginosa* (Figure 2, panels C and D). The bottle was supplied during March 18–27, 2013, first opened in September 2013, and removed from use when sampling was performed in December 2013.

### Electron Microscopy

**Figure 3:**
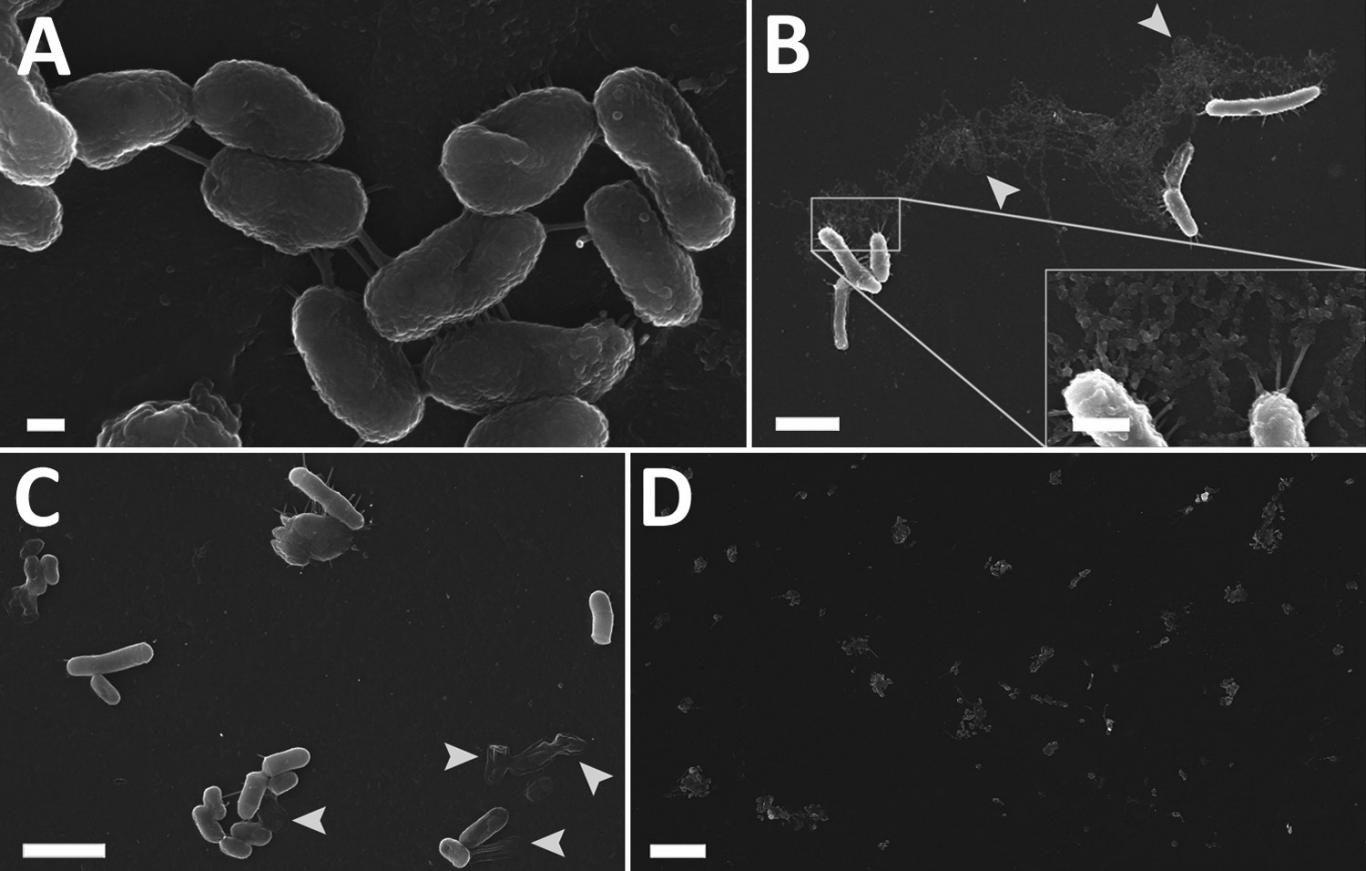
Scanning electron micrographs of internal plastic surface of contaminated irrigation fluid bottle implicated in a 2012–2013 cutaneous melioidosis cluster in the temperate southern region of Western Australia. A–C) Bacilli tethered to each other and to the surface by short peritrichous or polar adhesions (A, C) and occasionally by fibrillary material (B), which appeared to have a globular structure at higher magnification. Decayed cells were common (arrows). D) Clusters of cells were regularly dispersed over the surface. Scale bars indicate 2 μm (A); 200 nm (B and C); 500 nm (B, inset); and 10 μm (D).

Scanning electron microscopy of the contaminated wound irrigation fluid bottle confirmed extensive bacterial colonization of the inner surfaces. The density of bacterial colonization varied depending on the location analyzed within the bottle. Few bacteria were at the neck of the bottle, many were on the sides of the bottle, and the highest concentration was at the base of the bottle. Bacteria were tethered by short adhesions (Figure 3, panel A) and were occasionally associated with fibrillary material that had a globular structure at high magnification (Figure 3, panel B inset). Decayed bacteria were common (Figure 3, panel C, arrows), and although duplex cells were observed, healthy dividing cells were uncommon. Numerous clusters and microcolonies were observed, particularly at the base of the bottle, which also showed a greater proportion of decayed cells and more extensive extracellular adhesions than the other samples (Figure 3, panel D). The plastic washer inside the bottle lid was covered in a mature biofilm.

## Discussion

Recent events in the continental United States highlight the ability of *B. pseudomallei* to breach ecologic or biologic boundaries (*26*–*30*). A review of U.S. state and territory cases identified 3 persons with culture-positive melioidosis in the absence of relevant travel to melioidosis-endemic regions and concluded with a recommendation that physicians and healthcare workers should be more aware of the disease (*31*). A lack of familiarity with this bacterial species in an unusual clinical setting, such as occurred in the cluster we describe here, can cause difficulty in identifying the primary source. The previous Western Australia cluster occurred in a very different setting, a remote community in the tropical north of the state (*7*). Only 1 other melioidosis cluster has been reported in temperate Western Australia; that cluster was attributed to livestock transported from a melioidosis-endemic region (*6*). The melioidosis cluster we describe here was notable for its occurrence in urban Western Australia, which is not considered to be endemic for melioidosis, and for its association with a contaminated wound care product.

We recovered *B. pseudomallei* isolates with the same multilocus ST from 6 melioidosis patients in 1 postal district and excluded another 2 cases of melioidosis from our investigation on the basis of clinical features, travel history, biogeographic bacterial clade, and MLST genotype. Isolation of *B. pseudomallei* of the same ST from contaminated wound irrigation fluid explains the 5 cases in 2013 because the bottle of wound irrigation fluid was used without replacement throughout this period. Although the manufacturer’s instructions advise that the bottle should be discarded within 24 hours of opening and label the fluid as single-use, it is common practice to use such large volumes of fluid as a stock during wound care procedures, providing decanting is conducted as a no-touch procedure. This melioidosis cluster highlights the public health risks of such a practice. Because the contaminated wound irrigation fluid bottle had not yet been received when the 2012 patient had wound care at the same health facility, the wound irrigation fluid probably was not contaminated before opening. We note that the connection between the January 2012 case and the September–December 2013 cases remains unexplained. Given that other patients received wound care during this period without evidence of cutaneous melioidosis, the existence of an earlier contaminated bottle with subsequent transfer to the contaminated bottle is improbable. Possible explanations for this interval include an undetected past case of chronic, unresolved cutaneous melioidosis with multiple introductions of *B. pseudomallei* into medical products or an external environmental reservoir common to the 2012 patient and the first or first few 2013 patients. The previous report of nosocomial melioidosis in Australia identified environmental *B. pseudomallei* that was biochemically similar to the clinical isolates, but that report lacked the strength of molecular epidemiology evidence (*10*). All previous reports of nosocomial melioidosis come from locations in the tropics (*8*–*12*). 

The melioidosis cluster we report represents an unusual healthcare-associated outbreak in a temperate suburban setting. *B. pseudomallei* was not isolated from any other solution or environmental sample from the facility. It is not clear whether the count of *B. pseudomallei* found in the contaminated irrigation fluid was the result of an initial seeding event with subsequent bacterial growth or by gross contamination without further growth. Previous in vitro studies indicate that *B. pseudomallei* will tolerate a wide range of nutrient-free aqueous environments (*32*), and survival of 10^7^ CFU/mL *B. pseudomallei* in sterile distilled water over 16 years has been reported (*33*). Electron microscopy showed complex colonization patterns and extensive bacterial adhesion, consistent with long-term bacterial colonization. The initial seeding event probably included sufficient nutrients to support bacterial proliferation. The senescent bacteria we observed could reflect a larger original bacterial population introduced to the bottle. Although not healthcare-associated, 2 cases of cutaneous melioidosis attributed to a contaminated hand wash solution noted in another report further highlight the potential for contamination and subsequent transmission of *B. pseudomallei* in an occupational setting (*34*).

We investigated cause and effect in this laboratory outbreak investigation by using a set of stringent rules for emerging infectious disease causality (*35*), establishing the laboratory evidence to link a series of cutaneous infections in a geographically restricted cluster, identifying a probable source, and introducing early environmental controls. We used a combination of molecular epidemiology, microscopy, and culture-based bacteriologic methods to identify and study a point source for healthcare-associated infection in this investigation of a cluster of cutaneous melioidosis cases in temperate southwest Australia. We obtained circumstantial evidence that bacterial contamination combined with incorrect use of wound irrigation fluid to form a chain of events necessary for subsequent infection. The contaminated wound irrigation fluid is a plausible vehicle for infection, but uncertainty exists about its essential role in the case of patient A. Although we halted the series of infections by removing the source of *B. pseudomallei* infection from use, we have not yet been able to identify the initial environmental reservoir. Therefore, the long duration of the previous temperate Western Australia cluster leads us to expect additional sporadic cases in the area over an extended period and represents a continuing public health risk (*4*). However, we have not identified any other cases of cutaneous melioidosis in the same area or ST-1112 infections elsewhere during the subsequent 12 months of laboratory-based surveillance.

Although the inoculum of *B. pseudomallei* causing human cutaneous infection was measured, the circumstances of wound contamination in this cluster did not allow us to determine the probability of subsequent dissemination from an already infected wound. Additional questions raised are 1) whether pouring contaminated wound irrigation fluid limited infection to an already damaged epidermis without generating sufficient aerosol for pulmonary infection, and 2) whether others in this suburban community were exposed to *B. pseudomallei* ST-1112 without clinical consequences. Specific aspects of virulence phenotype, genetics, and circumstance that resulted in the notable absence of pneumonia or septicemic infection are the focus of further study.
